# A novel system with robust compatibility and stability for detecting *Sugarcane yellow leaf virus* based on CRISPR-Cas12a

**DOI:** 10.1128/spectrum.01149-24

**Published:** 2024-08-09

**Authors:** Ting Wang, Anzhen Li, Hong Zhao, Qibin Wu, Jinlong Guo, Helei Tian, Jingwen Wang, Youxiong Que, Liping Xu

**Affiliations:** 1 Key Laboratory of Sugarcane Biology and Genetic Breeding, Ministry of Agriculture and Rural Affairs, National Engineering Research Center for Sugarcane, College of Agriculture, Fujian Agriculture and Forestry University, Fuzhou, China; 2 National Key Laboratory for Tropical Crop Breeding, Institute of Tropical Bioscience and Biotechnology, Chinese Academy of Tropical Agricultural Sciences, Sanya, China; South China Agricultural University, Guangzhou, China

**Keywords:** *Sugarcane yellow leaf virus*, MIRA, CRISPR-Cas12a, sugarcane

## Abstract

*Sugarcane yellow leaf virus* (SCYLV) can reduce sugarcane productivity. A novel detection system based on reverse transcription-multienzyme isothermal rapid amplification (RT-MIRA) combined with CRISPR-Cas12a, named RT-MIRA-CRISPR-Cas12a, was developed. This innovative approach employs crude leaf extract directly as the reaction template, streamlining the extraction process for simplicity and speed. Combining RT-MIRA and CRISPR-Cas12a in one reaction tube increases the ease of operation while reducing the risk of aerosol contamination. In addition, it exhibits sensitivity equivalent to qPCR, boasting a lower detection limit of 25 copies. Remarkably, the entire process, from sample extraction to reaction completion, requires only 52–57 minutes, just a thermostat water bath. The result can be observed and judged by the naked eye.

**IMPORTANCE:**

Sugarcane yellow leaf disease (SCYLD) is an important viral disease that affects sugarcane yield. There is an urgent need for rapid, sensitive, and stable detection methods. The reverse transcription-multienzyme isothermal rapid amplification combined with CRISPR-Cas12a (RT-MIRA-CRISPR-Cas12a) method established in this study has good specificity and high sensitivity. In addition, the system showed good compatibility and stability with the crude leaf extract, as shown by the fact that the crude extract of the positive sample could still be stably detected after 1 week when placed at 4°C. RT-MIRA-CRISPR-Cas12a, reverse transcription polymerase chain reaction (RT-PCR), and reverse transcription-quantitative polymerase chain reaction (RT-qPCR) were used to detect SCYLV on 33 sugarcane leaf samples collected from the field, and it was found that the three methods reached consistent conclusions. This Cas12a-based detection method proves highly suitable for the rapid on-site detection of the SCYLV.

## INTRODUCTION

Sugarcane (*Saccharum* spp. hybrids) contributes significantly to global sugar production, constituting 80% of the world’s total. Sugarcane yellow leaf disease (SCYLD), caused by the *Sugarcane yellow leaf virus* (SCYLV), is transmitted through sugarcane seed stalks and insect vectors (1), resulting in yield losses of up to 50% (2). The characteristic symptom of the disease is severe yellowing of the midrib on the abaxial surface of leaves, which usually occurs while the leaves are still green. As the disease progresses, areas of discoloration extend outward from the midrib and wilt from the leaf tips to the leaf base (3, 4). However, distinguishing SCYLV phenotypic symptoms from yellow leaf syndrome caused by sugarcane yellows phytoplasma (SCYP) is challenging due to high similarity (5–7). At the same time, factors such as sugarcane cultivations, nutrient imbalances, and maturity process can also induce leaf yellowing (4), complicating phenotypic recognition. Relying solely on leaf phenotypical symptoms to determine whether the SCYLV is infected may lead to misjudgment. Therefore, establishing a detection technology with specificity, stability, and sensitivity is crucial for the accurate determination for pathogen.

Currently, the molecular technologies used for SCYLV detection are mainly reverse transcription polymerase chain reaction (RT-PCR) and reverse transcription-quantitative polymerase chain reaction (RT-qPCR). Although both methods have good stability and high sensitivity (8–11), they have limitations for the detection of large quantitative samples and point-of-care testing (POCT), because the RNA extraction, PCR, and qPCR operations require well-trained personnel, more expensive and sophisticated instruments and equipment, and are time-consuming. Recently, based on isothermal amplification, RT-loop-mediated isothermal amplification (RT-LAMP) and RT-recombinase polymerase amplification (RT-RPA) have been developed for the detection of SCYLV (12, 13). Among them, the RT-LAMP technique established by Anandakumar et al. can detect 10 pg of positive RNA samples, which is 10 times more sensitive than RT-PCR (12). The reaction time of the RT-RPA technology established by Feng et al. is only 20 minutes, and the sensitivity can detect at least 10^3^-fold diluted cDNA (223 pg/µL of positive RNA) (13). Besides, the multienzyme isothermal rapid amplification (MIRA) developed based on RPA technology has also been applied to pathogen detection, such as Severe Acute Respiratory Syndrome Coronavirus 2 (SARS-CoV-2) and *Staphylococcus aureus* (14, 15). The benefits of isothermal amplification offset the insufficient RT-PCR and RT-qPCR, including the elimination of the need for thermal cycle equipment and a quick detection time, and also avoiding the nonspecific amplification and false positives of the RT-LAMP and RT-RPA (16, 17).

The method of clustered regularly interspaced short palindromic repeats-CRISPR-associated proteins (CRISPR-Cas) was developed in 2017, which was first applied in the systematic detection of pathogenic bacteria (18). In the Cas enzyme system, both Cas12a and Cas13a are suitable for nucleic acid detection (19, 20). Cas12a is a crRNA-guided DNA endonuclease that cleaves the target DNA strand when it forms a complex with guide RNA and Cas12a. This activates the collateral cleavage ability of Cas12a, which cleaves the target fragment. Studies have found that when the collateral cleavage ability of Cas12a is activated, it can non-specifically cut any ssDNA sequence in the system, a phenomenon known as reverse cleavage (21, 22). Once ssDNA with indicator signals is placed in the detection system at this time, the presence of target nucleic acid can be determined through the indicator signals. The discovery of this feature has led to the application of CRISPR-Cas12a in nucleic acid detection. It was firstly developed for detecting *human papillomavirus* (HPV) (22). Later, the system was also applied to SARS-CoV-2 detection (23). Combined with RPA, the detection needs 50 minutes from sample extraction, and its sensitivity is between 1 and 10 copies (23). Due to the high sensitivity, specificity, and low cost of the CRISPR-Cas12a detection system, it is very suitable for the detection of plant pathogens (24–28). For example, Jiao et al. (26) employed this system in conjunction with RPA technology to visually identify five distinct apple virus types, including *Apple necrotic mosaic virus* (ApNMV), *Apple stem pitting virus* (ASPV), *Apple stem grooving virus* (ASGV), *Apple chlorotic leaf spot virus* (ACLSV), and *Apple scar skin viroid* (ASSVd). The detection threshold for ASPV and ASGV is 250 virus copies per reaction, whereas the threshold for other viruses is 2,500 virus copies. The total reaction time is 60 minutes. Subsequently, the visual detection of *Maize chlorotic mottle virus* (MCMV) was also reported (27, 28). The basic idea of these detection systems is to firstly amplify the target sequence by nucleic acid isothermal amplification to obtain a large number of templates, and then use the CRISPR-Cas12a to cut the signal probe, achieving the detection of low-load samples. However, these systems involve two-step reactions, and it is necessary to open the reaction tube and transfer the liquid, which increases the complexity of the detection procedure and is very prone to aerosol contamination (21). Some improvements have been proposed for the one-tube reaction, including the spatial isolation (29, 30), using glycerol for transient isolation (31), the light activation (32), the microfluidic technology (33, 34), and the suboptimal protospacer adjacent motifs (PAM) region crRNA method (35).

**Fig 1:**
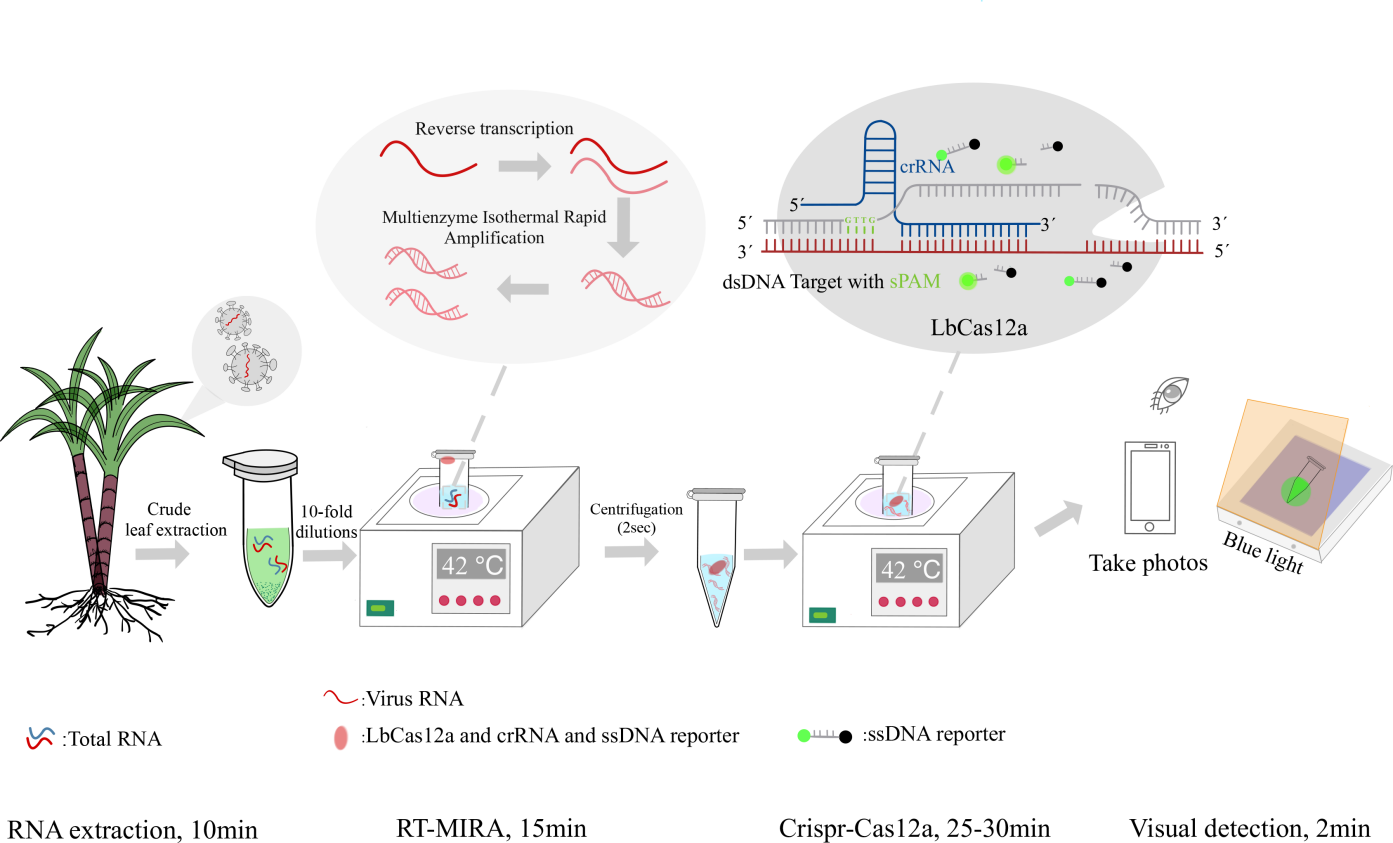
Flowchart of RT-MIRA-CRISPR-Cas12a visualization method for detection of *Sugarcane yellow leaf virus*.

In this study, we combined RT-MIRA with CRISPR-Cas12a to establish a one-tube detection method for SCYLV (Fig. 1). The CRISPR-Cas12a system is first added to the tube wall or cap using a spatial isolation method. Then, the MIRA system is allowed to react for 15 minutes, and the mixture of the MIRA system and CRISPR-Cas12a is quickly mixed using centrifugation. Using suboptimal PAM for crRNA design effectively avoids the decrease in sensitivity caused by the competition between the two systems. A new technology for detecting SCYLV was established.

## MATERIALS AND METHODS

### Sample collection and RNA extraction

The 33 sugarcane leaves were collected from the sugarcane germplasm garden of Fujian Agriculture and Forestry University in Fuzhou, Fujian, China, including Liucheng 05-136 (LC05-136), Guitang 42 (GT42), Guitang 49 (GT49), Yunzhe 08-1609 (YZ08-1609), Yacheng 01-48 (YC 01-48), Yunrui 03-175 (YR03-175), Yuetang 93-159 (YT93-159), ROC 22, LA purple, Badila, Q200, CP49-50, CP63-588, FN 14-13, FN 14-23, FN 14-25, FN 14-65, FN 14-71, FN 14-83, FN 14-85, FN 14-98, FN 14-128, FN 14-151, FN 14-159, FN 14-171, FN 14-209, FN 14-213, FN 14-228, FN 14-276, FN 14-398, FN 14-307, FN 14-325, and FN 14-400. Leaf (~100 mg) RNA was extracted using a Trozol kit (Magen Biotechnology, Guangzhou, China), and the integrity of RNA was detected by 1% agarose gel electrophoresis. The concentration was determined by spectrophotometer (Thermo Fisher Scientific, Waltham, MA, USA) and was stored at −80°C, and then the cDNA chain was synthesized by reverse transcription kit and was stored at −20°C for later use.

Preparation of RNA crude extract was conducted by alkaline polyethylene glycol extraction method (26, 36). Weighing 15 mg sugarcane leaves, and after grinding in liquid nitrogen, 300 µL extract (6% PEG200 [Sangon Biotech, Shanghai, China] and 20 mM NaOH [Macklin, Shanghai, China]) was added. They were thoroughly mixed and placed at room temperature for 5–10 minutes, and the RNA crude extract was obtained. It can be diluted 10 times for immediate detection or stored at 4°C.

### Construction of SCYLV standard plasmid

PCR was used to amplify the target band from the positive cDNA sample provided by the National Engineering Research Center for Sugarcane of Fujian Agriculture and Forestry University. The target band was a partial fragment of the SCYLV-*CP* gene with a length of 588 bp. The primer information is as follows: SCYLV-F: 5′-CCGCTCACGAAGGAATGTCA-3′/SCYLV-R:5′-GGAGCGTCGCCTACCTATT-3′ (37). Reaction system: 1.0 µL positive cDNA (800 ng/µL), 15 µL 2× Taq PCR StarMix (GenStar, Beijing, China), 1.0 µL each forward and reverse primer, and sterile water to make up to 30 µL. PCR reaction program: 94°C; 2 minutes; 35 cycles of 94°C, 30 seconds, 55°C, 30 seconds, 72°C, 1 minute; 72°C, 10 minutes. Construction of the pUC57-SCYLV plasmid: the putative target fragment was recovered, inserted into the pUC57 vector (Sangon Biotech, Shanghai, China), and sent to Shanghai Sangon Engineering Co., Ltd, for sequencing. The previous method (38) was used to determine the plasmid DNA concentration and to calculate the plasmid copy number. The calculation formula is as follows: plasmid copy number (copies/µL) = (6.02 × 10^23^copy/mol) × (*N* × 10^−9^) / (M × 660 g/mol/dp), where *N* (ng/µL) is the plasmid concentration and M is the plasmid length in base pairs.

### Design of MIRA primers and crRNA

Here in our study, 10 SCYLV complete genome sequences were downloaded from GenBank with accession numbers of GU190159, GU570008, AM072756, GU327735, JF925154, GU570007, KF477092, NC_000874, AF157029, and MF622078. Multiple sequence alignment was performed to screen out the conserved fragments in the full genome of SCYLV using DNAMAN 8 (https://www.lynnon.com/index.html). Primer Premier 5 was then used to design the MIRA primer (https://www.premierbiosoft.com) according to the instructions of the MIRA kit (Weifang Amp-Future Biotech, Shandong, China). The primer is MIRA-F: 5′-TCACGAAGGAATGTCAGAAGACGCGCTAAC-3′/MIRA-R: 5′-TTCGGTCCGAATTTGAGGATCCCGGTTGAG-3′.

The suboptimal PAM can reduce the early cleavage efficiency of Cas12a and reduce competition with isothermal amplification for one-tube detection (35). Therefore, in this study, the PAM selected is suboptimal PAM (GTTG). The crRNA sequence is 5′-UAAUUUCUACUAAGUGUAGAUGAGGAAACGCUGUGCGAGGA-3′, which was synthesized by Nanjing GenScript Biotechnology Company (GenScript, Nanjing, China).

### MIRA assay

The reaction system contained 14.7 µL A buffer (WLRB8207KIT, Weifang Anpu Future Biotechnology Co., Ltd., Shandong Province, China), 6.05 µL nuclease-free water, 1.0 µL (10 pmol/µL) MIRA-F, 1.0 µL (10 pmol/µL) MIRA-R, 1.25 µL B buffer (Weifang Anpu Future Biotechnology Co., Ltd., Shandong Province, China), and 1.0 µL template. First, twice the volume of the above solution (excluding the template) was added to the reaction tube containing freeze-dried enzyme particles, shaken up and down to mix, and then divided into two equal parts, and 1.0 µL of template was added to each part for reaction. Finally, the reaction tubes were placed in 42°C water and incubated for 30 minutes. When the incubation was completed, 5.0 µL of amplified product was combined with 1.0 µL of 6× loading buffer, and the mixture was incubated for 5 minutes in a 56°C water bath. Using 1% agarose gel, electrophoretic experiments were conducted, and the gel results were observed using a chemiluminescence imager.

### Establishment of RT-MIRA-CRISPR-Cas12a visualization detection system

The reaction system created by combining the MIRA with the CRISPR-cas12a is shown in Table 1. To begin, the A buffer, MIRA-F, MIRA-R, B buffer, and nuclease-free water of the MIRA system were combined at the bottom of a 200-µL centrifuge tube. The Cas12a (Tolobio, Shanghai, China), 10 HOLMES buffer 1 (Tolobio, Shanghai, China), ssDNA reporter (5′-FAM-TTATTAT-BHQ2-3′) (Sangon Biotech, Shanghai, China), and crRNA were then mixed and added to the centrifuge tube wall or cap (mixed into multiple tubes and then divided into aliquots). Finally, the reaction template RNA was added to the MIRA system at the bottom of the centrifuge tube, placed at 42°C for 15 minutes to react, and then centrifuged briefly to mix the MIRA and CRISPR-Cas12a systems before placing in the Quant Studio 3 (Thermo Fisher Scientific, Waltham, MA, USA) instrument at 42°C. After 30 minutes, the real-time fluorescence value was measured. After the reaction, the results were examined with a blue light (470 nm B2042, UE Landy, Suzhou, China) and a mobile phone (Xiaomi 10s, Xiaomi, Beijing, China).

**TABLE 1:** CRISPR-Cas12a combined with MIRA in the one-tube reaction system

System composition	Usage amount (µL)
Cas12a (10 pmol/µL)	0.125
10× HOLMES buffer 1	1.0
ssDNA reporter (10 pmol/µL)	0.25
crRNA (70 ng/µL)	0.175
A buffer	14.7
MIRA-F (10 pmol/µL)	1.0
MIRA-R (10 pmol/µL)	1.0
B buffer	1.25
Template	1.0
Nuclease-free water	9.5
Total volume	30

### Sensitivity examination

Following confirmation of the copy number of the plasmid constructed in the manner as described above (2.7 × 10^10^ copies/μL in this study), the plasmid was diluted to 1.0 × 10^3^ copies/μL and then diluted in a 10-fold gradient to prepare dilutions of each concentration ranging from 1.0 × 10^3^ copies/μL to 1.0 × 10^0^ copies/μL, plus the dilutions of 50 copies/μL and 25 copies/μL. A total of six different plasmid dilution concentrations were used for the sensitivity test of the three different methods. The MIRA-CRISPR-Cas12a reaction system is the same as that in Table 1. The reaction method and results are recorded as described above. The PCR reaction primers are YLS111 : 5′-TCTCACTTTCACGGTTGACG-3′/YLS462 : 5′-GTCTCCATTCCCTTTGTACAGC-3′ (39). The reaction system and reaction procedures are the same as described above. The qPCR reaction primers are F: 5′-GCGTTCAACAATGGCTTACTC-3′/R: 5′-GACTTTCTTGGCGTTCCTCTT-3′ (40), and the reaction system is 1.0 µL of forward and reverse primers, 2× PerfectStart Green qPCR SuperMix 15.0 µL, template 1.0 µL, and sterile water were added to 30 µL. The reaction procedure was based on the kit instructions (AQ602, TransGen Biotech, Beijing, China), and the annealing temperature was set to 60°C.

### Specificity determination of RT-MIRA-CRISPR-Cas12a method

To validate the specificity of the RT-MIRA-CRISPR-Cas12a one-tube method, the RNA from sugarcane leaves respectively infected by *Sugarcane mosaic virus* (SCMV), *Sorghum mosaic virus* (SrMV), and *Sugarcane streak mosaic virus* (SCSMV) were used as samples. Additionally, the RNA extracted from virus-free and SCYLV-infected sugarcane leaves respectively served as a virus-absent negative control and positive control, and a water template was employed as a blank control. The reaction system was identical to that shown in Table 1. The reaction method and results were documented in the manner as described above.

### Accuracy verification for RT-MIRA-CRISPR-Cas12a method

After diluting the RNA crude extracts of 33 field samples by 10 times, 2.0 µL of the diluted solution was used for the RT-MIRA-CRISPR-Cas12a one-tube detection. The reaction components are the same as in Table 1, and the reaction procedures are as described above. The RNA extracted by the Torzol method was used to reverse transcribe and synthesize cDNA, whereas PCR and qPCR detection were performed to mutually verify the detection results. The reaction procedures for PCR and qPCR are the same as described above. After the PCR reaction, 5.0 µL of the PCR product was used for electrophoresis on 1.0% agarose gel, and the results were recorded.

### Validity test of RNA crude extract

After grinding 15 mg of the SCYLV-infected sugarcane leaves in liquid nitrogen, 300 µL of extraction solution (6% PEG200 and 20 mM NaOH) was added and mixed thoroughly, and left at room temperature for 5–10 minutes to obtain the crude extracts, which was considered as the 1× dilution (10^0^). Furthermore, 10-fold serial dilutions 10^−1^, 10^−2^, 10^−3^, and 10^−4^ were made for testing. Then, the 10^−1^ times crude extract prepared and stored at 4°C was tested on the 3rd, 5th, and 7th days, respectively, to observe the validity period of the crude RNA extracts.

## RESULTS

### A positive plasmid pUC57-SCYLV was verified by sequencing

The base sequence of the constructed pUC57-SCYLV plasmid was completely consistent with the expected sequence of the *CP* gene fragment (588 bp) (Fig. S1). Besides, the plasmid concentration detected by the ultra-micro-volume spectrophotometer was 96.6 ng/µL, the total length of pUC57-SCYLV plasmid was 3,298 bp, and the pUC57-SCYLV plasmid copy number = (6.02 × 10^23^) × (96.6 ng/µL × 10^−9^) / (3,298 × 660), that is, the plasmid copy number was 2.7 × 10^10^ copies/µL.

### The RT-MIRA-CRISPR-Cas12a one-tube detection method was successfully developed

**Fig 2:**
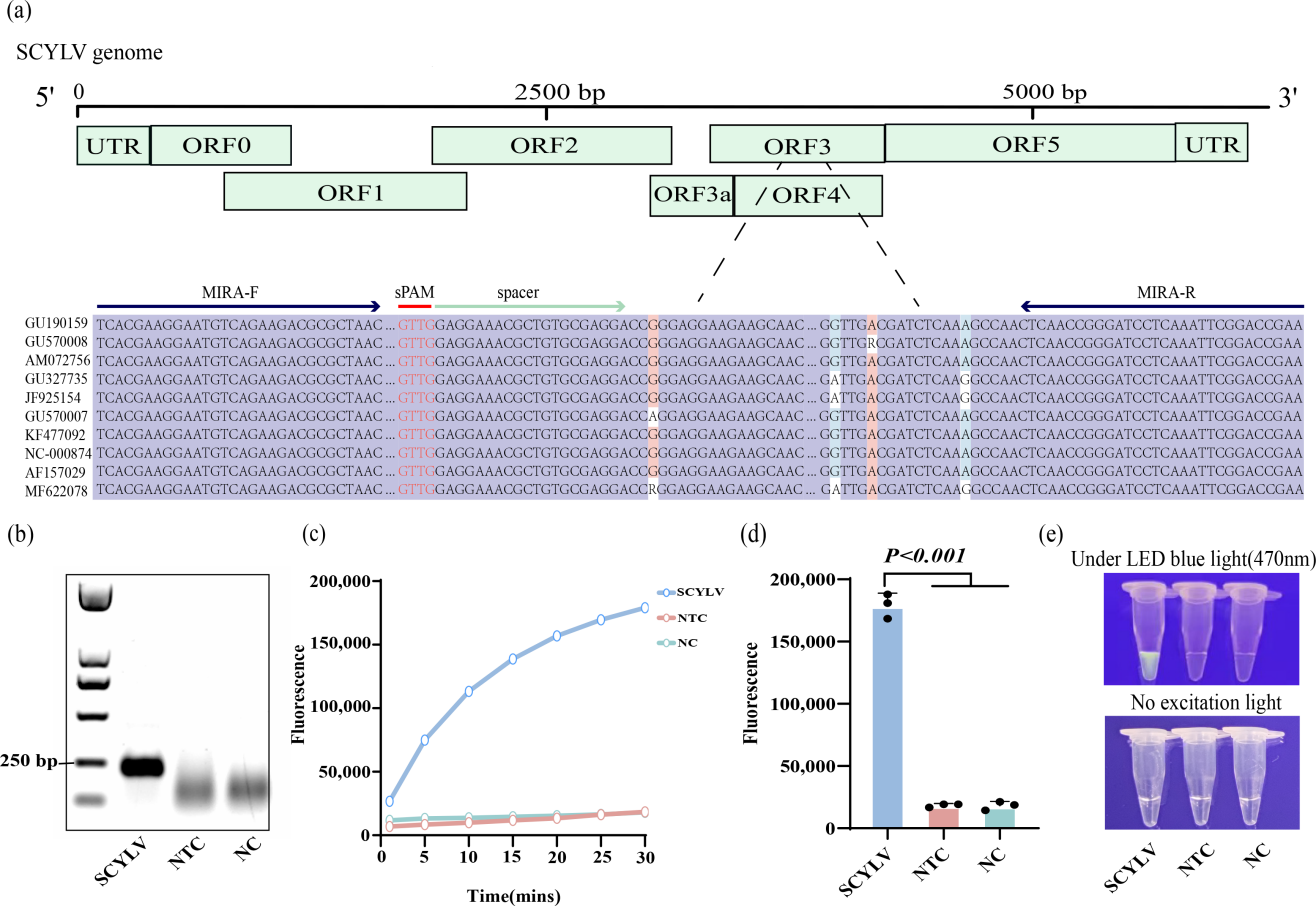
One-tube detection of SCYLV by CRISPR-Cas12a combined with MIRA amplification. (**a**) The genome map of SCYLV with the detailed sequence information of the designed primers and crRNA spacer sequence. Ten sequences of different isolates of SCYLV were obtained from GenBank and were aligned by the DNAMAN8 program. (**b**) MIRA amplification electropherogram. SCYLV, positive RNA template; NTC, negative control with virus-free sugarcane seedlings as template; NC, blank control with sterile ddH_2_O as template. (**c**) After 15 minutes of MIRA amplification, the real-time fluorescence of the CRISPR cutting probe was recorded. Three replicates were run (*n* = 3), and data were plotted using averages. (**d**) End-point fluorescence. The error bars represent the means ± SD (*n* = 3). Unpaired two-tailed *t*-test was used to analyze the statistical significance. (**e**) Visualization of the results at the end of the reaction.

Based on the alignment of 10 whole genome sequences of SCYLV from different isolates (Fig. 2a), a region exhibiting a highly conserved sequence, with an identity of 99.16%, was identified within the ORF3 coding frame (Fig. S2). Subsequently, primers were designed in this region for MIRA isothermal amplification. Remarkably, the primer pair, demonstrating robust amplification capabilities, produced a band corresponding to the 221 bp anticipated target fragment (Fig. 2b). Concurrently, the crRNA sequence was designed within this target fragment (Fig. 2a).

After the incubation of centrifuge tubes containing the MIRA and the CRISPR-Cas12a systems, fluorescence values were measured. A conspicuous and rapid increase in fluorescence intensity was observed in the positive RNA reaction tube, accompanied by a distinct fluorescence signal (Fig. 2c). In stark contrast, the fluorescence values of the negative control and blank control were markedly lower, rendering them imperceptible to the naked eye (Fig. 2c and e). Furthermore, quantitative analysis of the fluorescence values further underscored the efficacy of the developed RT-MIRA-CRISPR-Cas12a one-tube detection system. Compared to both the negative control and the blank control, the positive reaction tube exhibited significantly higher fluorescence values, a distinction demonstrated in the quantitative representation presented in Figure 2d.

### The MIRA-CRISPR-Cas12a detection system can stably detect 25 copies of the plasmid

**Fig 3:**
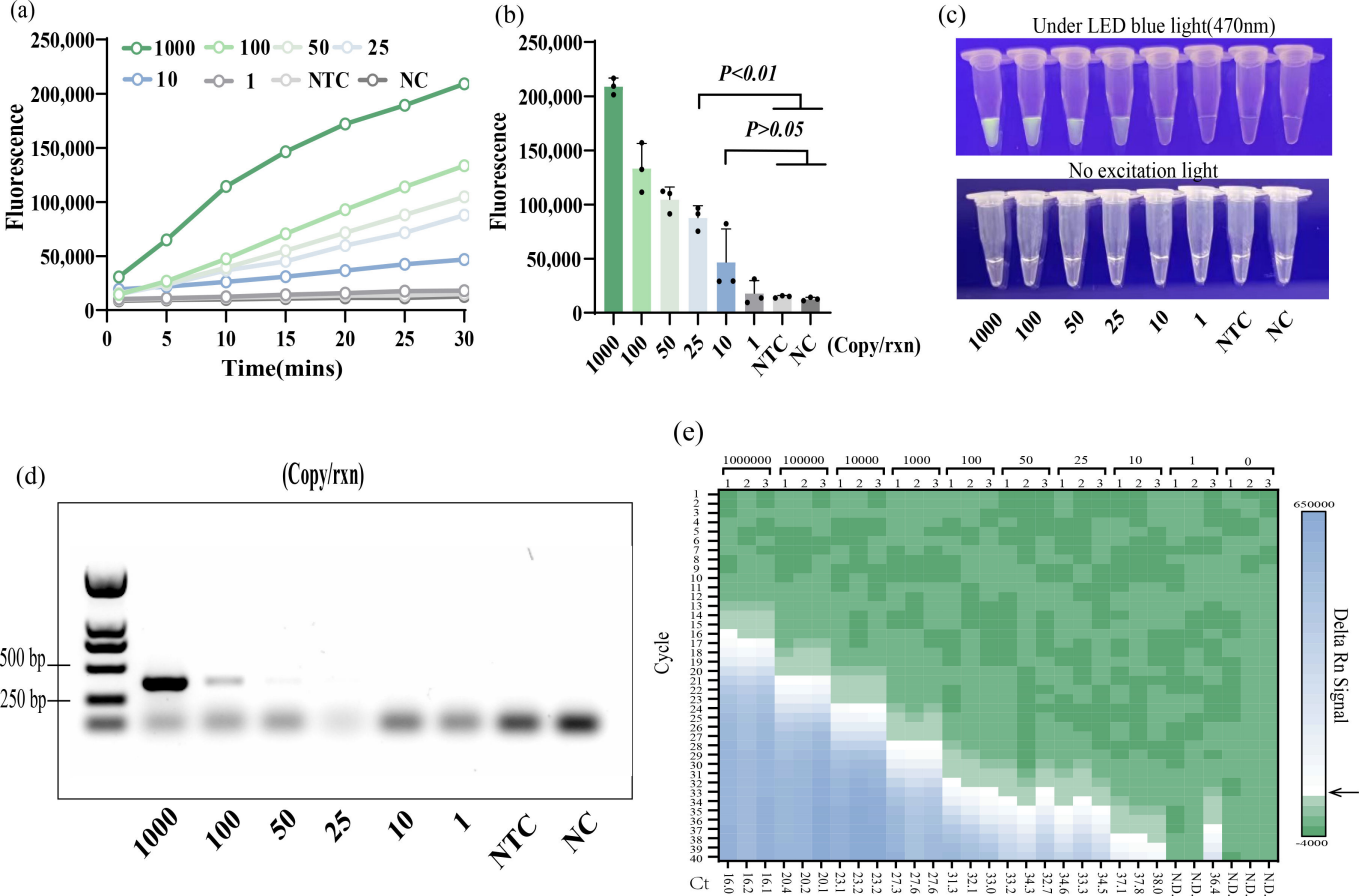
Sensitivity detection. (**a**) After 15 minutes of MIRA amplification, the real-time fluorescence of the CRISPR cutting probe was recorded. Three replicates were run (*n* = 3), and data were plotted using averages. 1,000, 100, 50, 25, 10 and 1, respectively, represent plasmids containing 1,000, 100, 50, 25, 10 and 1 copy in each reaction. NTC, negative control with virus-free sugarcane seedlings as template; NC, blank control with sterile ddH_2_O as template. (**b**) End-point fluorescence. The error bars represent the means ± SD (*n* = 3) from replicates. Unpaired two-tailed *t*-test was used to analyze the statistical significance. (**c**) Visualization of the results at the end of the reaction. (**d**) Sensitivity results of PCR reaction. (**e**) Heatmap indicates fluorescence signal generated by qPCR in sensitivity test; the abscissa represents the Ct value of the reaction. The black arrow indicates the threshold point of the reaction.

It can be seen from the fluorescence curve in Figure 3a that the fluorescence value of the MIRA-CRISPR-Cas12a detection system increases with the prolongation of the reaction time, and the positive plasmid can be stably detected in a reaction tube containing 25 copies of the plasmid. Its end-point fluorescence value was significantly higher than the negative and blank controls (Fig. 3b). The weak fluorescence can be seen with the naked eye in the 10-copy reaction tube (Fig. 3c). However, the detection results are unstable, and the fluorescence value is not significantly different from the negative and blank controls (Fig. 3b). This indicates that the system has reached its minimum detection limit, and although faint fluorescence is visually observed, it lacks the robustness required for reliable detection, at the lower level of copy number.

PCR and qPCR sensitivity tests were conducted on six different plasmid dilutions. The results revealed that at 100 copies/reaction, the PCR products had obvious bands, and when it was further reduced to 50 copies/reaction, the bands were barely visible (Fig. 3d). The sensitivity test of qPCR showed the Ct value changes in the gradients 10^6^–10^3^ copies/reaction, indicating that the dilution is effective, and the minimum detectable limit for Ct values below 35 is 25 copies, because at lower concentrations of 10 copies and 1 copy, the Ct values consistently exceed 35 (Fig. 3e). To confirm this result, the reaction with a Ct value greater than 35 was repeated, and it was found that the results obtained with 10 copies and 1 copy were unstable, so we believed that the sensitivity of qPCR was 25 copies.

### There was excellent specificity of the RT-MIRA-CRISPR-Cas12a detection system

**Fig 4:**
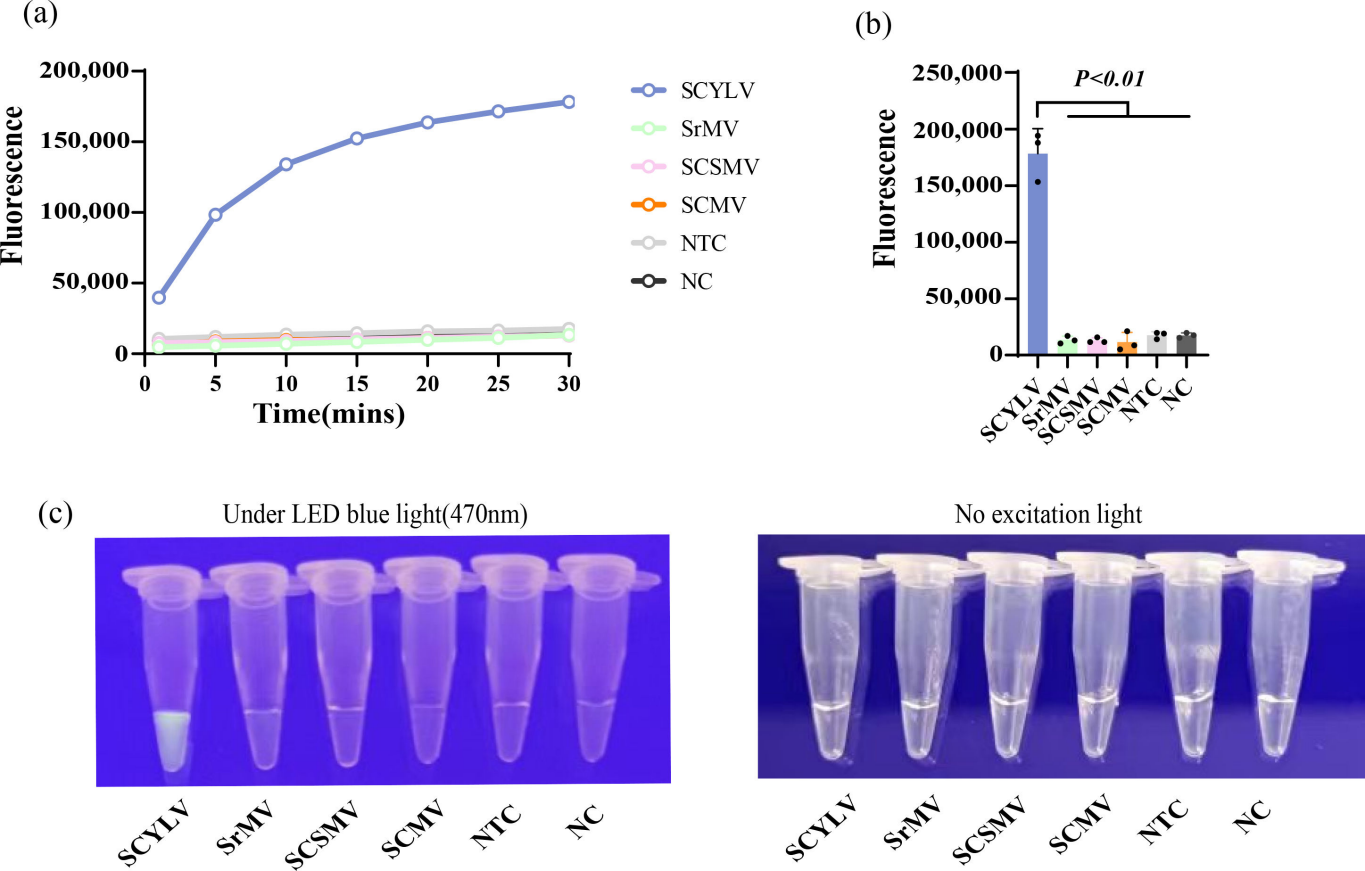
Specificity of RT-MIRA-CRISPR-Cas12a detection system. (**a**) After 15 minutes of MIRA amplification, the real-time fluorescence of the CRISPR cutting probe was reco rded. Three replicates were run (*n* = 3), and data were plotted using averages. SCYLV, positive RNA template; NTC, negative control with virus-free sugarcane seedlings as template; NC, blank control with sterile ddH_2_O as template. (**b**) End-point fluorescence. The error bars represent the means ± SD (*n* = 3) from replicates. Unpaired two-tailed *t*-test was used to analyze the statistical significance. (**c**) Visualization of the results at the end of the reaction.

The RT-MIRA-CRISPR-Cas12a system was used to detect sugarcane leaf RNA samples containing different viruses (SCMV, SrMV, SCSMV), and no visible fluorescence was observed in the above samples, the same as that in the blank control. In contrast, distinct fluorescence was observed in the leaf sample containing SCYLV (Fig. 4c). At the same time, the test tube with SCYLV exhibited a significantly higher end-point fluorescence value compared to the other five tubes (Fig. 4b). Judging from the changes in real-time fluorescence data, the curve containing SCYLV rises rapidly as time increases, but the fluorescence values of the remaining five tubes do not change significantly as time increases (Fig. 4a). These indicate that the RT-MIRA-CRISPR-Cas12a detection system has a good specificity.

### The application of RT-MIRA-CRISPR-Cas12a detection method was successful

**Fig 5:**
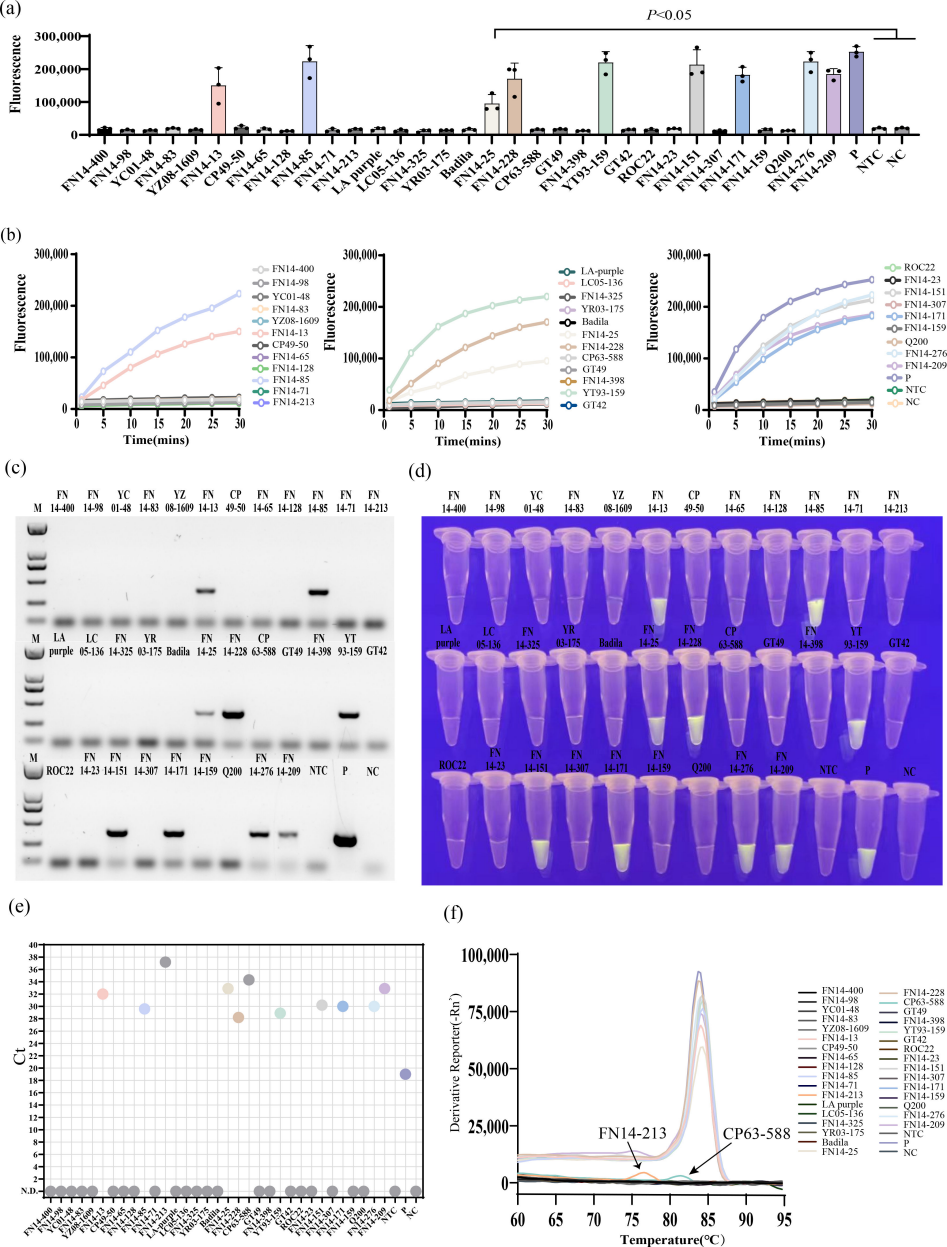
The detection results of MIRA-CRISPR-Cas12a technology are consistent with PCR and qPCR. (**a**) After 15 minutes of MIRA amplification, the real-time fluorescence of the CRISPR cutting probe was recorded. Three replicates were run (*n* = 3), and data were plotted using averages. P, positive template; NTC, negative control with sugarcane detoxification seedlings as template; NC, blank control with sterile ddH_2_O as template. (**b**) End-point fluorescence. The error bars represent the means ± SD (*n* = 3) from replicates. Unpaired two-tailed *t*-test was used to analyze the statistical significance. (**c**) PCR test results of the 33 samples. (**d**) Visualization of the results at the end of the reaction. (**e**) qPCR test results of the 33 samples. The abscissa is the name of each sample, and the ordinate is the Ct value of each sample. (**f**) The melt curve plot of the 33 sample qPCR reactions.

A total of 33 sugarcane leaf RNA samples were subjected to PCR analysis, and it was found that nine of the samples amplified the target fragments, with a positive detection rate of 27.3% (9/33), namely, FN14-13, FN14-85, FN14-25, FN14-228, YT93-159, FN14-151, FN14-171, FN14-276, and FN14-209. After qPCR testing, among the 33 samples, FN14-13, FN14-85, FN14-213, FN14-25, FN14-228, CP63-588, YT93-159, FN14-151, FN14-171, FN14-276, and FN14-209 all have Ct values, among which the Ct values of FN14-213 and CP63-588 are greater than 35, and the remaining samples are between 28.2 and 32.9 (Fig. 5e). Furthermore, based on the melt curve plot of 33 sample qPCR reactions, FN14-13, FN14-85, FN14-213, FN14-25, FN14-228, YT93-159, FN14-151, FN14-171, FN14-276, and FN14-209 have a single melt peak, whereas FN14-213 and CP63-588 do not have a typical single melt peak (Fig. 5f). Combining the Ct value and the amplification peak, it was determined that the qPCR detection results of FN14-213 and CP63-588 were negative, and the Ct value that appeared in the detection was speculated to be caused by primer dimers. Therefore, the qPCR detection results of 33 sugarcane leaf samples were consistent with the PCR detection (Fig. 5c, d, and f).

The above 33 RNA crude extracts were directly used for RT-MIRA-CRISPR-Cas12a detection. The results showed that the fluorescence value of nine samples increased rapidly as the reaction time increased (Fig. 5b). At the end of the reaction, fluorescence could be observed with the naked eye in nine samples (Fig. 5d), and the end-point fluorescence value was significantly higher than the negative and blank controls (Fig. 5a). During comparison, these nine fluorescent samples were completely consistent with the positive detection samples of PCR and qPCR. However, the fluorescence values among the three technical biological replicates in each of the mentioned nine positive sugarcane clones were different. It is thus speculated that despite the consistent sampling volume of 15 mg for each technical biological replicate, the variation may stem from differing virus content in the prepared crude leaf RNA extracts. However, it does not impact the determination of positive detection results. The end-point fluorescence images of the other two replicates are shown in Figure S3 and S4.

### The crude leaf RNA extract is still valid after a week of storage at 4°C

**Fig 6:**
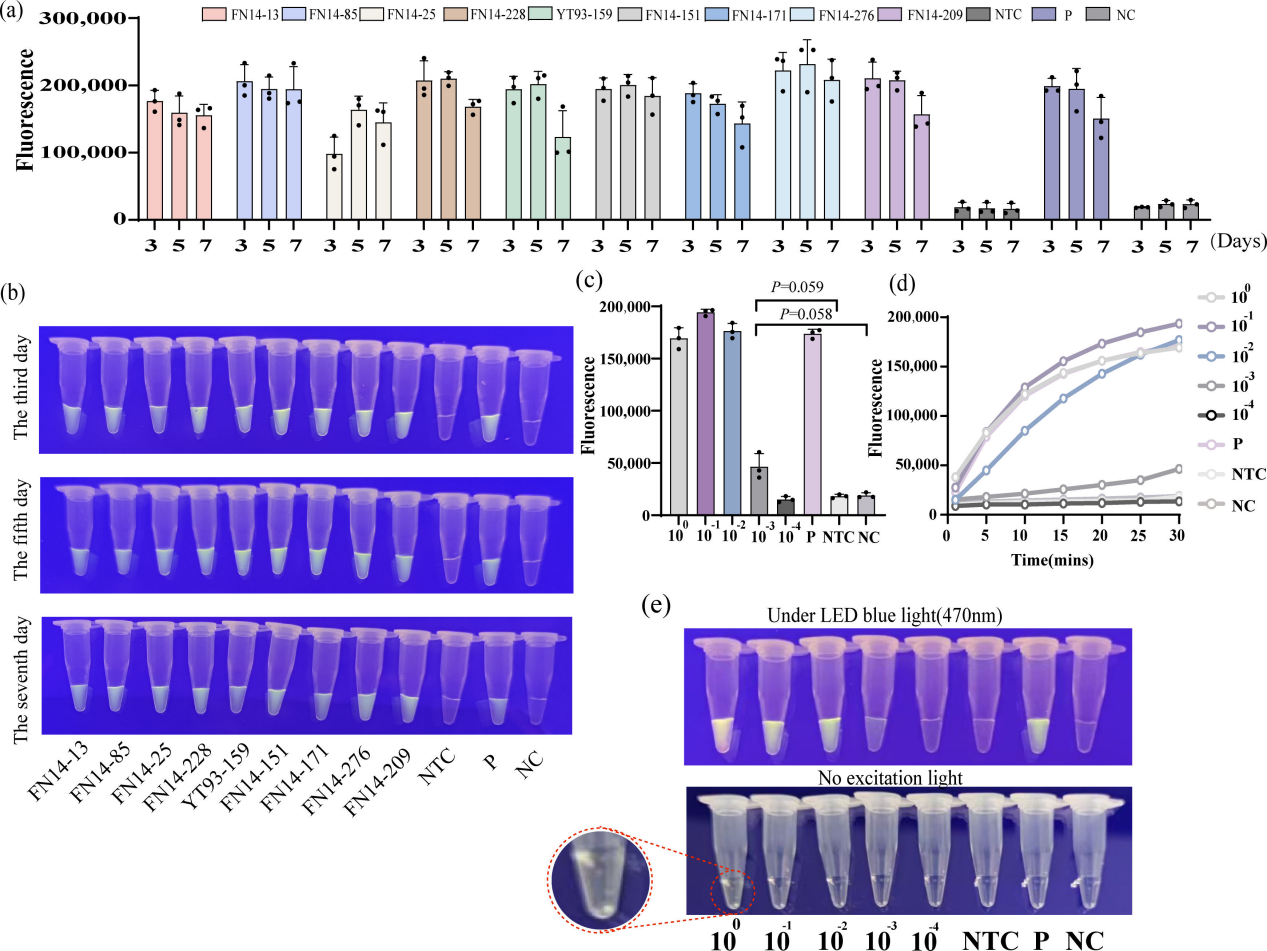
RNA crude extract expiration date and effectiveness. (**a**) The RT-MIRA-CRISPR-Cas12a system was used to detect the nine positive crude extracts stored at 4°C for 3, 5, and 7 days, and the end-point fluorescence value was recorded. P, positive crude extracts; NTC, negative control with sugarcane detoxification seedlings as template; NC, blank control with sterile ddH_2_O as template. (**b**) Visual diagram of the reaction after being placed at 4°C for 3, 5, and 7 days, respectively. (**c**) End-point fluorescence. The error bars represent the means ± SD (*n* = 3) from replicates. Unpaired two-tailed *t*-test was used to analyze the statistical significance. The 10^−1^–10^−4^ are the crude extracts diluted according to 10 times gradient. (**d**) After 15 minutes of MIRA amplification, the real-time fluorescence of the CRISPR cutting probe was recorded. Three replicates were run (*n* = 3), and data were plotted using averages. (**e**) Visual results after the reaction of crude extracts with different dilution ratios. The red circle is a partial enlargement of 10°.

The system was used to test the crude RNA extracts of SCYLV-positive leaves of nine sugarcane varieties used for the RT-MIRA-CRISPR-Cas12a one-tube detection after storing at 4°C for 3, 5, and 7 days. The fluorescence could be observed with the naked eye in all the positive samples, and the detected fluorescence values were higher than the negative and blank controls (Fig. 6a and b), indicating that the crude extract can be stored at 4°C and that the detection results are stable within the tested period (7 d).

In the randomly selected sugarcane hybrid FN14-209, obvious fluorescence can be observed when the crude leaf RNA extract was diluted to 10^−1^ and 10^−2^ (Fig. 6e), and the fluorescence value was significantly higher than the negative and blank controls. However, when the dilution was continued to 10^−3^, there was no significant difference in fluorescence values among the mentioned three samples (Fig. 6c). It is worth noting that the fluorescence value of 10^0^ is lower than the value of 10^−1^ (Fig. 6c and d). This occurs most probably because the natural color of the high-concentration crude extract is green (Fig. 6e). Nevertheless, it appears red under blue light, and the color of the leaves itself will interfere with the recording of fluorescence values.

## DISCUSSION

The symptoms of SCYLD caused by SCYLV and yellow leaf syndrome caused by SCYP are similar, and it is difficult to quickly distinguish them phenotypically (41). Meanwhile, the phenotypic symptoms of the SCYLD are more obvious at the maturation stage in general, and the most obvious and typical phenotypic symptom of this disease is the yellowing of the leaf midvein, which is similar to the phenotypic characteristics of leaves at the stage of maturity in sugarcane (3, 4). Therefore, the development of a portable detection method is realistic for field SCYLV monitoring and supervision of virus-free seedlings, which promotes early treatment and prevents the rapid spread of the virus. Commonly used detection methods, such as PCR and qPCR, are similar to the method developed in this study. Additionally, qPCR technology is often hailed as the “gold standard” for pathogen detection (42), but it can only be completed in the laboratory, which is not conducive to rapid detection and not so convent to confirm the test results by the naked eye in the field (11). LAMP and RPA can react under constant temperature conditions, which is simple to operate and not restricted by professional environments and instruments. The LAMP detection method for SCYLV, as established by Anandakumar et al. (12), exhibits sensitivity levels intermediate between PCR and qPCR. The lower detection limit is 10 pg RNA, whereas PCR can only reach 100 pg, which is 10 times higher than PCR and falls slightly below the sensitivity of qPCR (12). The sensitivity of the RPA technique to detect SCYLV is slightly inferior. The sensitivity can only detect 10^3^ times diluted cDNA (223 pg/µL positive RNA) (13), which is 10 times lower than PCR, but the advantage of RPA over LAMP lies in its simpler primer design, operating at a milder temperature, typically around 37℃. In contrast, LAMP requires 65℃, imposing higher reaction conditions. Additionally, LAMP primer design is intricate, and multiplex detection poses greater challenges than with RPA. In addition to molecular diagnosis, enzyme-linked immunosorbent assay (ELISA) and tissue-blot immunoassay (TBIA) have been employed for the detection of SCYLV (43, 44). These two immunological-based approaches are well suited for rapidly screening a substantial number of samples, facilitating short-term, large-scale detection. However, the sensitivity of immunological detection is contingent upon the quality of the antibody utilized. The production of specific antibodies in the initial stages is tightly associated with elevated costs and time requirements (45).

In the past, all those conventional molecular detection methods mentioned for SCYLV need sample pre-processing and involve a laborious RNA extraction process (8–13). Here in our study, an RT-MIRA-CRISPR-Cas12a system for detecting SCYLV was developed. This study uses direct reaction of crude leaf extracts, which has the advantages of short time and simplified operation. At the same time, the combination of CRISPR-Cas12a effectively reduces the possibility of false positives. Its efficiency is evident in the overall process from leaf extraction to obtaining results, ranging between 52 and 57 minutes, a short sample reaction time of 45 minutes, and the ability to detect 25 copies of the positive plasmid (0.83 copies/μL), indicating that the sensitivity is four times greater than that of PCR and comparable to qPCR. In contrast, conventional SCYLV detection through PCR and qPCR necessitates 60–120 minutes (8, 46), which is longer than the developed method here. Of course, results visualization is also undoubtedly an advantage over PCR and qPCR.

Surprisingly, the response times in this study were shorter or comparable to other studies using CRISPR systems to detect other pathogens in other species. For instance, Lei et al. (2023) employed RPA combined with CRISPR-Cas12 for the rapid detection of the MCMV, and the reaction time is 50 minutes and the sensitivity is 2.5 *CP* gene copies (28). Similarly, Xiong et al. (2020) used the same method to detect the new coronavirus, with a reaction time of 50 minutes and a sensitivity of 1–10 copies (23). At the same time, Lei et al. (2022) used the same method to detect *Leptosphaeria maculans* (*L. maculans*) within 45 minutes with a LOD of 4.7 genomic DNA copies (47). Not only that, Shin et al. (2021) detected citrus scab successfully with a 45-minute reaction, with a lower detection limit of 10^−6^ ng (1 fg) template DNA (48). It is worth mentioning that Singh et al. (2023) achieved a more rapid detection, that is, they only needed 30 minutes to get the result, and the detection limit of MPOX virus was 1 copy/μL (49). In addition, Wang et al. (2023) showed the ability to detect 2.26 copies/μL of SrMV plasmid with a short reaction time of 30 minutes (50). However, these two reports adopt two steps, whereas a one-step system developed in this study is more convenient.

Additionally, our method exhibits good specificity. SCYLV is an RNA virus with a nucleotide length of approximately 6,000 bp (51). Based on the alignment of 10 whole genome sequences of SCYLV from different isolates, a region exhibiting a highly conserved sequence, with an identity of 99.16% in this region, was identified within the ORF3 coding frame (Fig. S2). The ORF3 coding frame is also the region where primers are designed by RT-PCR and RT-qPCR (10, 52); therefore, this study carried out MIRA primer design in this region. Due to the possibility of nonspecific amplification with isothermal amplification alone (53, 54), this experiment integrated CRISPR-Cas12a with MIRA to enhance the specificity of the system. Nevertheless, it is essential to highlight that the crRNA target sequence is generally 20–30 bp (22, 55, 56). In the case of plants like sugarcane, characterized by large genomes, it is also crucial to assess whether the target sequence might interact with the sugarcane genome, potentially leading to false positives.

To enhance suitability for field testing, this study referenced the methodology by Wang et al., incorporating reverse transcription, the MIRA system, and the CRISPR-Cas12a system for reactions conducted within a centrifuge tube (29, 30). Compared with the two-step detection, this one-step method not only simplifies the operation steps but also reduces the risk of aerosol contamination (22), mostly caused by opening the lid of the centrifuge tube. Concurrently, the direct utilization of crude leaf extracts in the reaction developed in this system obviates the need for fussy nucleic acid extraction, which significantly improved convenience compared to traditional PCR detection methods. Additionally, our method facilitates result visualization and eliminates the requirement for an electrophoresis step (28). The study ascertained that the crude extract, when stored at 4°C, maintains stability in test results over 1 week. This observation underscores the commendable compatibility with crude extracts.

Based on the above, our method provides a new way for on-site detection of SCYLV. By utilizing this technology, valuable insights are provided into the detection of various pathogens affecting sugarcane. The versatility of this approach is evident as it allows the design of different crRNA and amplification primers tailored to different nucleic acid sequences, establishing a valuable reference for broader applications in pathogen detection.

## SUPPLEMENTAL MATERIAL

Supplemental material spectrum.01149-24-s0001.pdf Fig. S1 to S4.

ASM does not own the copyrights to Supplemental Material that may be linked to, or accessed through, an article. The authors have granted ASM a non-exclusive, world-wide license to publish the Supplemental Material files. Please contact the corresponding author directly for reuse.
