# Supplementary material for: A novel system with robust compatibility and stability for detecting Sugarcane yellow leaf virus based on CRISPR-Cas12a
Source: Microbiol Spectr. 2024 Aug 9;12(9):e01149-24. doi: 10.1128/spectrum.01149-24 (PMC11370245; doi:10.1128/spectrum.01149-24)
Supplement: Supplemental material — Fig. S1 to S4. [file spectrum.01149-24-s0001.pdf]

```
Expected_amplification_of_product  CCGCTCACGAAGGAATGTCAGAAGACGCGCTAACCGTCGTAGACAGACTCGGCCAGTGGT   60
Sequencing_results                 CCGCTCACGAAGGAATGTCAGAAGACGCGCTAACCGTCGTAGACAGACTCGGCCAGTGGT   60
Consensus                          ccgctcacgaaggaatgtcagaagacgcgctaaccgtcgtagacagactcggccagtggt

Expected_amplification_of_product  CGTGGTCCGGGCTCCCCCAGGACCTAGACGAGTACGACGACGTAGAGCACGTGTTGGAGG  120
Sequencing_results                 CGTGGTCCGGGCTCCCCCAGGACCTAGACGAGTACGACGACGTAGAGCACGTGTTGGAGG  120
Consensus                          cgtggtccgggctcccccaggacctagacgagtacgacgacgtagagcacgtgttggagg

Expected_amplification_of_product  AAACGCTGTGCGAGGACCGGGAGGAAGAAGCAACCGGGATGTTCTCACTTTCACGGTTGA  180
Sequencing_results                 AAACGCTGTGCGAGGACCGGGAGGAAGAAGCAACCGGGATGTTCTCACTTTCACGGTTGA  180
Consensus                          aaacgctgtgcgaggaccgggaggaagaagcaaccgggatgttctcactttcacggttga

Expected_amplification_of_product  CGATCTCAAAGCCAACTCAACCGGGATCCTCAAATTCGGACCGAACTTATCTCAGTACGC  240
Sequencing_results                 CGATCTCAAAGCCAACTCAACCGGGATCCTCAAATTCGGACCGAACTTATCTCAGTACGC  240
Consensus                          cgatctcaaagccaactcaaccgggatcctcaaattcggaccgaacttatctcagtacgc

Expected_amplification_of_product  AGCGTTCAACAATGGCTTACTCAAAGCCTACCATGAGTATAAAATCACAAGTCTCACTAT  300
Sequencing_results                 AGCGTTCAACAATGGCTTACTCAAAGCCTACCATGAGTATAAAATCACAAGTCTCACTAT  300
Consensus                          agcgttcaacaatggcttactcaaagcctaccatgagtataaaatcacaagtctcactat

Expected_amplification_of_product  TCAGTATAACTCATGCTCCTCCGACGCAACTCCAGGTGCAATCGCACTTGAAGTGGATAC  360
Sequencing_results                 TCAGTATAACTCATGCTCCTCCGACGCAACTCCAGGTGCAATCGCACTTGAAGTGGATAC  360
Consensus                          tcagtataactcatgctcctccgacgcaactccaggtgcaatcgcacttgaagtggatac

Expected_amplification_of_product  ATCCTGCTCCCAAACAACAACAGGCTCCAAGATTACTAGCTTCCCCGTCAAGAGGAACGC  420
Sequencing_results                 ATCCTGCTCCCAAACAACAACAGGCTCCAAGATTACTAGCTTCCCCGTCAAGAGGAACGC  420
Consensus                          atcctgctcccaaacaacaacaggctccaagattactagcttccccgtcaagaggaacgc

Expected_amplification_of_product  CAAGAAAGTCTTCCCGGCCCCCTTCATCAGGGGGAAAGATTTCATGACTACGTCAGCTGA  480
Sequencing_results                 CAAGAAAGTCTTCCCGGCCCCCTTCATCAGGGGGAAAGATTTCATGACTACGTCAGCTGA  480
Consensus                          caagaaagtcttcccggcccccttcatcagggggaaagatttcatgactacgtcagctga

Expected_amplification_of_product  CCAGTTTTGGTTGCTGTACAAAGGGAATGGAGACTCGAGCCTAGCAGGACAATTCGTCTG  540
Sequencing_results                 CCAGTTTTGGTTGCTGTACAAAGGGAATGGAGACTCGAGCCTAGCAGGACAATTCGTCTG  540
Consensus                          ccagttttggttgctgtacaaagggaatggagactcgagcctagcaggacaattcgtctg

Expected_amplification_of_product  CCGATTTGAATGCCTTTTCCAGAATCCCAAATAGGTAGGCGACGCTCC              588
Sequencing_results                 CCGATTTGAATGCCTTTTCCAGAATCCCAAATAGGTAGGCGACGCTCC              588
Consensus                          ccgatttgaatgccttttccagaatcccaaataggtaggcgacgctcc
```

Supplementary Fig S1. Sequencing results of the pUC57-SCYLV plasmid.

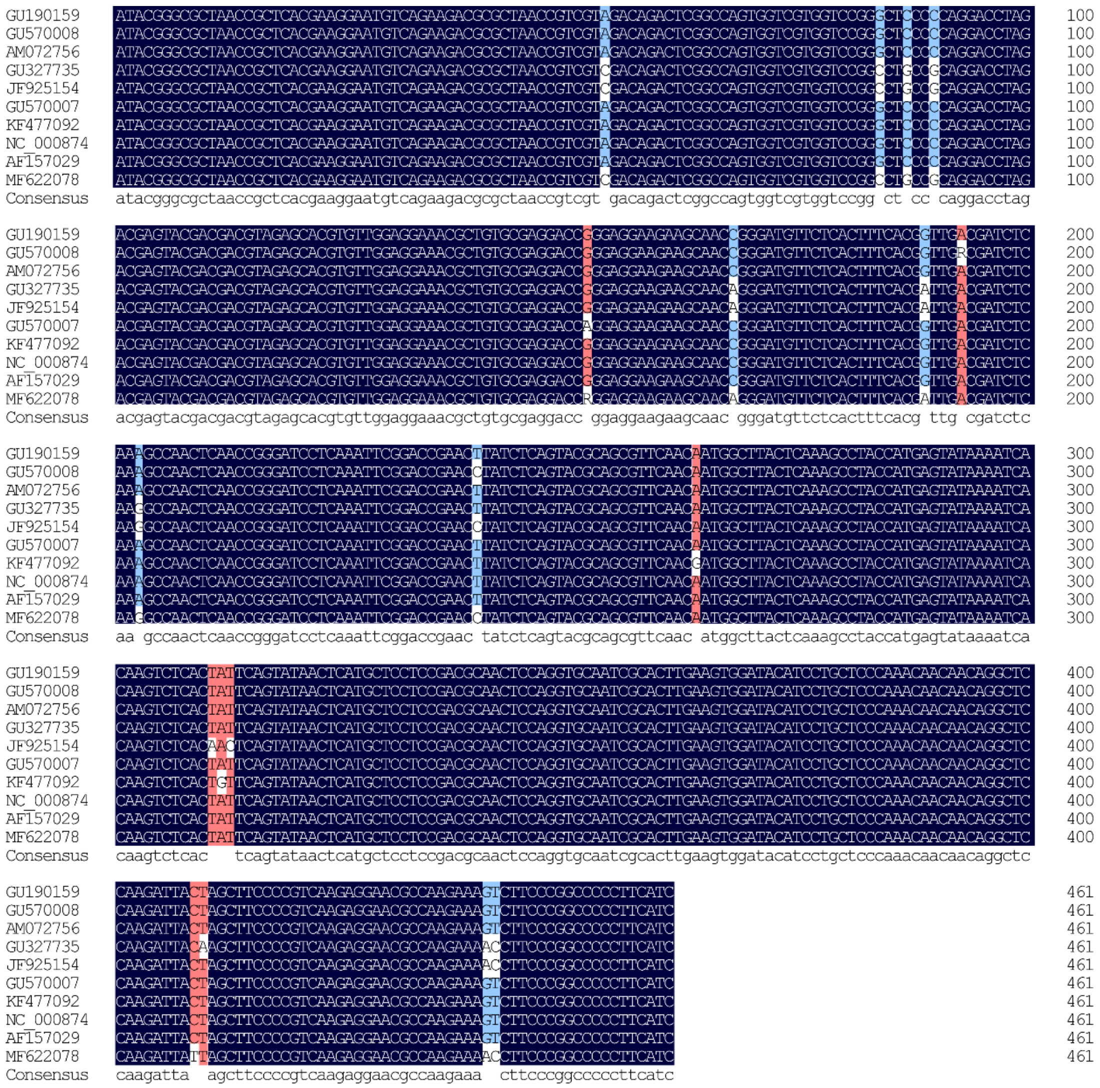

Supplementary Fig S2. A region showing a high degree of sequence conservation was identified within the ORF3 coding frame, with an identity of 99.16%.

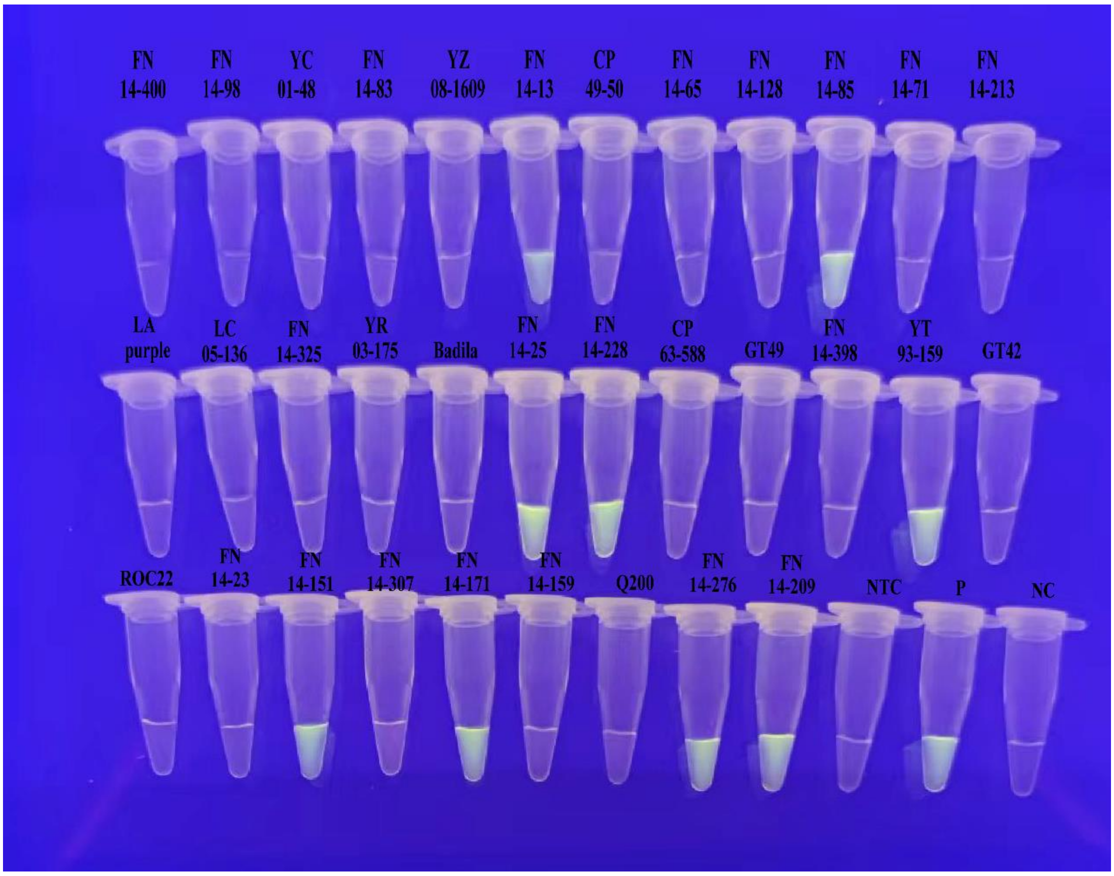

Supplementary Fig S3. Picture of the second repeated detection of 33 field samples using the RT-MIRA-CRISPR-Cas12a method.

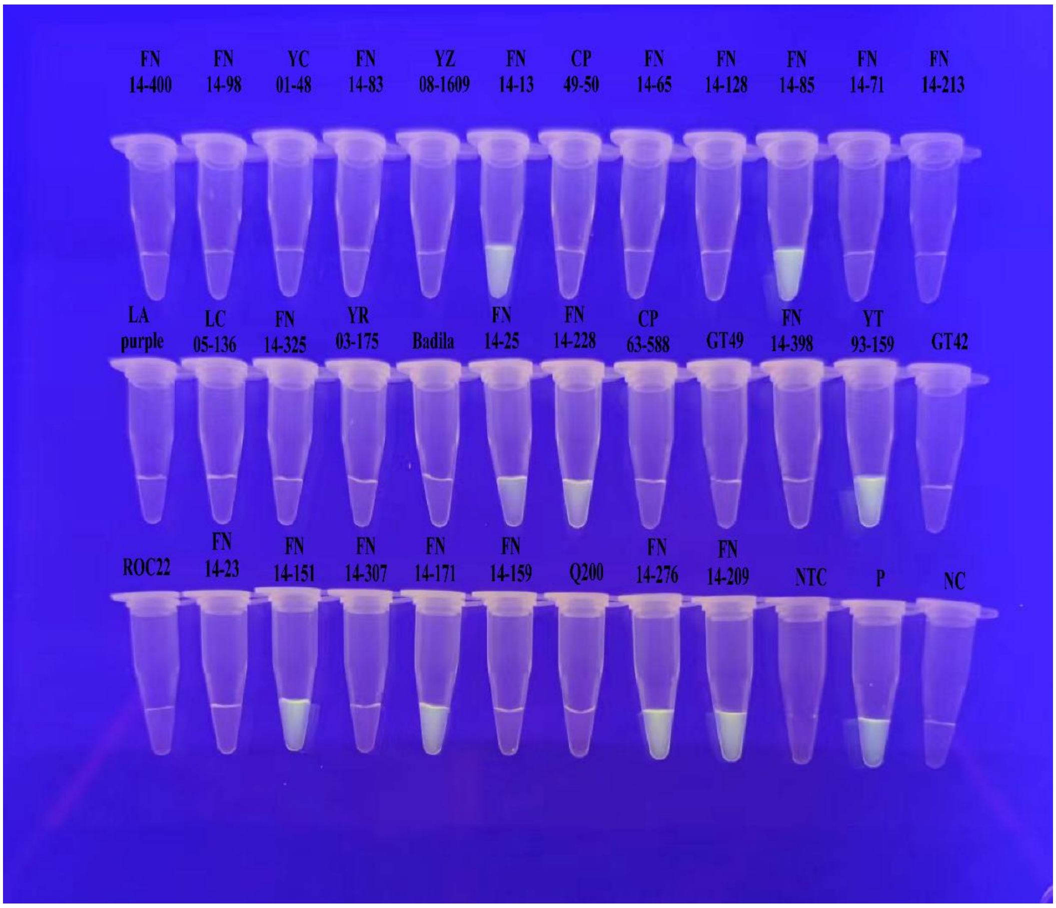

Supplementary Fig S4. Picture of the third repeated detection of 33 field samples using the RT-MIRA-CRISPR-Cas12a method.
